# A case of anti-CD320 antibody-positive cutaneous arteritis accompanied by multiple cranial nerve symptoms

**DOI:** 10.1093/rheumatology/keae528

**Published:** 2024-09-27

**Authors:** Akiko Hasegawa, Kazuki M Matsuda, Hirohito Kotani, Ai Kuzumi, Asako Yoshizaki-Ogawa, Ayumi Yoshizaki, Shinichi Sato

**Affiliations:** Department of Dermatology, The University of Tokyo Graduate School of Medicine, Tokyo, Japan; Department of Dermatology, The University of Tokyo Graduate School of Medicine, Tokyo, Japan; Department of Dermatology, The University of Tokyo Graduate School of Medicine, Tokyo, Japan; Department of Dermatology, The University of Tokyo Graduate School of Medicine, Tokyo, Japan; Department of Dermatology, The University of Tokyo Graduate School of Medicine, Tokyo, Japan; Department of Dermatology, The University of Tokyo Graduate School of Medicine, Tokyo, Japan; Department of Clinical Cannabinoid Research, The University of Tokyo Graduate School of Medicine, Tokyo, Japan; Department of Dermatology, The University of Tokyo Graduate School of Medicine, Tokyo, Japan

Rheumatology key message

- Our case indicated possible contribution of anti-CD320 antibody to cutaneous arteritis and cranial nerve impairments.

Dear Editor, Polyarteritis nodosa (PN) is a vasculitis affecting small- to medium-sized arteries, often presenting with systemic symptoms such as skin lesions, peripheral neuropathy and CNS manifestations [1]. Cutaneous PN (cPN), a subtype of PN confined to the skin [2], can occasionally require intensive immunosuppressive therapies [3]. In 2012, Revised International Chapel Hill Consensus Conference Nomenclature of Vasculitides documented cPN as ‘cutaneous arteritis’ (CA) [4]. We previously reported that approximately one-fourth of CA patients show serum positivity for anti-CD320 antibodies [5]. Here, we present a case of a 57-year-old male patient with CA, anti-CD320 antibodies, and multiple cranial nerve symptoms.

The patient presented with leg skin ulcers (Fig. 1A), which began 16 years ago and required oral prednisone (PSL). His medical history included appendicitis, cervical spondylosis, and cubital tunnel syndrome. A skin biopsy confirmed arteritis in the subcutaneous tissue (Fig. 1B), leading to a diagnosis of CA. Eight years ago, with PSL tapered to 3 mg/day, the ulcers reappeared, prompting referral to our clinic. Nerve conduction studies revealed sensory and motor impairment in the bilateral ulnar nerves. Blood tests showed elevated CRP and normal blood cell counts, with negative anti-nuclear and anti-neutrophil cytoplasmic antibodies. Proteome-wide autoantibody screening demonstrated higher anti-CD320 antibody levels compared with healthy controls (Fig. 1C and Supplementary Table 1, available at *Rheumatology* online) [5], confirmed by another protein array (Fig. 1D) [6]. Serum vitamin B12 (VB12) was elevated due to prior methylcobalamin (MetCbl) use. Cerebrospinal fluid analysis was normal. CT angiogram showed no arterial stenosis or aneurysm, and spine MRI indicated spinal stenosis.

**Figure 1.**
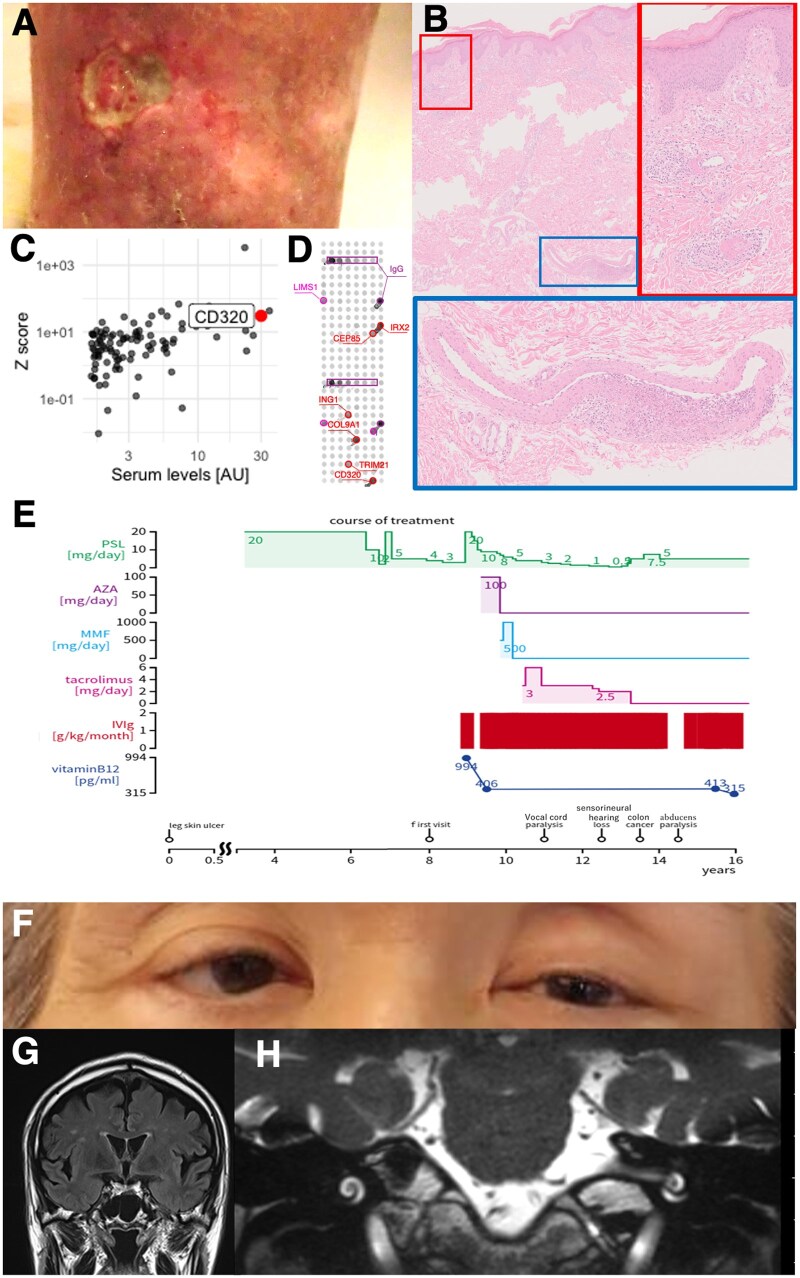
Clinical, histopathological and molecular features of the presented case. (A) Clinical pictures of his leg skin ulcer. (B) Histopathological images from a skin biopsy of his left ankle show evidence of vasculitis in the dermal vessels (red square) and in a subcutaneous arteriole (blue square). (C) A scatter plot illustrates autoantibody levels detected from his serum. X-axis shows absolute serum levels, while Y-axis represents *Z* scores compared with sex- and age-matched healthy controls (*n* = 12). AU: arbitrary unit. (D) A fluorescent image of a protein array shows positivity for serum anti-CD320 antibodies. IgG serves as a positive control. (E) A diagram illustrates the clinical course. AZA: azathioprine, MMF: mycophenolate mofetil. (F) A clinical picture of his eyes, demonstrating right esotropia. (G) A coronal section of a brain MRI using a fluid-attenuated inversion recovery (FLAIR) sequence shows no abnormal signals. (H) A horizontal section of the brainstem and inner ear on MRI images using a fast imaging employing steady-state acquisition (FIESTA) sequence shows no structural abnormalities

The case was considered refractory [3], leading to monthly IVIG and increased PSL to 20 mg/day (Fig. 1E). While skin ulcers improved, nerve conduction tests on his upper extremities showed no improvement. Additional immunosuppressants were tried but discontinued due to adverse events. MetCbl was halted due to lack of symptomatic improvement, leading to a decrease in serum VB12 levels. Three years later, the patient developed hoarseness and sensorineural hearing loss. Laryngoscopy revealed left vocal cord paralysis. Colon cancer was detected and treated, during which IVIG was halted, exacerbating leg ulcers. Moreover, the patient developed esotropia (Fig. 1F), confirmed as right abducens nerve palsy, but brain MRI ruled out structural abnormalities (Fig. 1G and H). Post-colectomy, he was maintained on PSL 5 mg/day and monthly IVIG, with partial improvement in leg skin ulcers, hoarseness, hearing loss and esotropia.

The aetiology of cranial nerve symptoms remained undetectable. While PN may cause ischaemic strokes due to intracranial small artery inflammation [1], brain MRI showed no such findings, supporting a diagnosis of CA rather than systemic PN [2]. Cranial nerve symptoms worsened with discontinuation of MetCbl and IVIG, improving with IVIG restart, suggesting a shared pathophysiology. In contrast, progressive ulnar nerve palsy during IVIG treatment may be attributed to cervical spondylosis or cubital tunnel syndrome.

The *CD320* gene encodes the transcobalamin receptor, mediating VB12 uptake into cells. Anti-CD320 antibodies can induce an autocrine loop of IL-6, a proinflammatory cytokine, and perivascular inflammation, suggesting their role in CA development [5]. Furthermore, anti-CD320 antibodies have been recently detected in a wider range of human disorders, indicating a broad spectrum of associated pathology. Anti-CD320 antibodies have been linked to CNS dysfunction of unknown origin, possibly inhibiting VB12 uptake by endothelial cells of the blood–brain barrier (BBB), leading to autoimmune VB12 central deficiency (ABCD) [7].

VB12 is crucial for haematopoiesis and myelin formation, and its deficiency can cause megaloblastic anaemia and neuropathy. CD320 knockout mice exhibit reduced VB12 in the CNS, causing cognitive disorders, brain atrophy and spinal cord demyelination [8]. While typical MRI findings of VB12 deficiency in the CNS include abnormal intensity in the posterior columns of the spine and the periventricular white matter, ABCD may not always appear on MRI [7]. Another key feature of ABCD is the sparing of peripheral nerves and the haematopoietic system, due to reliance on CD320-mediated VB12 uptake across the BBB in the CNS, with the low-density lipoprotein receptor serving as a backup in haematopoietic cells [7]. Consistently, anti-CD320 antibody-positive CA patients showed no peripheral neuropathy [5], and the current case had no haematopoietic abnormalities.

This case suggests that anti-CD320 antibodies may contribute to CA and neurological symptoms by inhibiting VB12 delivery to cranial nerve nuclei across the BBB. IVIG and MetCbl administration might be effective, but further research is needed to understand anti-CD320 antibody-associated syndromes and develop targeted therapeutic strategies.

## Supplementary Material

keae528_Supplementary_Data

## Data Availability

All data are incorporated into the article and its online supplementary material.

## References

[1] Matsuda KM , Koguchi A, Toyama T et al Concurrence of polyarteritis nodosa and multiple sclerosis. J Eur Acad Dermatology Venereol 2020;34:e188–91. 10.1111/jdv.16107 31769115

[2] Nakamura T , Kanazawa N, Ikeda T et al Cutaneous polyarteritis nodosa: revisiting its definition and diagnostic criteria. Arch Dermatol Res 2009;301:117–21. 18802715 10.1007/s00403-008-0898-2

[3] Matsuda KM , Yoshizaki A, Kotani H et al Development of a prediction model of treatment response in patients with cutaneous arteritis: insights from a cohort of 33 patients. J Dermatol 2021;48:1021–6. 33768589 10.1111/1346-8138.15868

[4] Jennette JC , Falk RJ, Bacon PA et al 2012 Revised International Chapel Hill consensus conference nomenclature of vasculitides. Arthritis Rheum 2013;65:1–11. 23045170 10.1002/art.37715

[5] Matsuda KM , Kotani H, Yamaguchi K et al Significance of anti-transcobalamin receptor antibodies in cutaneous arteritis revealed by proteome-wide autoantibody screening. J Autoimmun 2023;135:102995. 36724643 10.1016/j.jaut.2023.102995

[6] Matsuda KM , Yoshizaki A, Yamaguchi K et al Autoantibody landscape revealed by wet protein array : sum of autoantibody levels reflects disease status. Front Immunol 2022;13:1–14. 10.3389/fimmu.2022.893086 PMC9114879 35603173

[7] Pluvinage JV , Ngo T, Fouassier C et al Transcobalamin receptor antibodies in autoimmune vitamin B12 central deficiency. Sci Transl Med 2024;16:eadl3758. 38924428 10.1126/scitranslmed.adl3758 PMC11520464

[8] Arora K , Sequeira JM, Alarcon JM et al Neuropathology of vitamin B12 deficiency in the Cd320-/- mouse. FASEB J 2019;33:2563–73. 30303736 10.1096/fj.201800754RR PMC6338625

